# The Impact of Helmet Use on Injury Severity and Clinical Outcomes in E-Bike Riders

**DOI:** 10.7759/cureus.85153

**Published:** 2025-05-31

**Authors:** Graysen Pitcher, Monica Mendoza, Andrew McCague, Austin Henken-Siefken

**Affiliations:** 1 Medicine, Western University of Health Sciences, Pomona, USA; 2 Trauma and Acute Care Surgery, Desert Regional Medical Center, Palm Springs, USA; 3 Surgery, Desert Regional Medical Center, Palm Springs, USA

**Keywords:** bike, e-bike, electric bicycles, injury, protective benefits, trauma

## Abstract

Introduction

As electric bicycles (e-bikes) surge in popularity, understanding their impact on rider safety has never been more critical. This study presents a comprehensive analysis of helmet use trends, as well as the efficacy of helmet use in e-bike-related incidents. Key areas of analysis include demographics, injury severity, helmet use, and various clinical outcomes. With the significant rise in e-bike use in recent years, this study aims to evaluate trends in e-bike-related accidents and assess the role of helmet use in reducing injury severity and improving patient outcomes.

Methods

A retrospective analysis was performed using the National Trauma Data Standard (NTDS) from 2018 to 2023 to assess e-bike-related injuries. Patients were identified using 77 specific ICD-10 codes related to e-bike incidents, with 5,474 cases meeting the inclusion criteria. Demographic variables, injury markers, and clinical outcomes - including helmet use, hospital length of stay, ventilator days, Glasgow Coma Scale (GCS), and Injury Severity Score (ISS) - were analyzed. Statistical comparisons between helmeted and non-helmeted patients were conducted using Python 3.12.6, employing two-sided Welch’s t-tests and Student’s t-tests.

Results

A total of 7,018,732 patients were screened, yielding 5,474 cases of e-bike-related injuries between 2018 and 2023. The mean patient age was 41.1 ± 19.2 years, with 82% (4,493) being male. Demographic data revealed that 1,701 (31.1%) cases involved patients aged 40-60, followed by 1,674 (30.6%) cases aged 20-40, with helmet use rates of 29.5% (501) and 33.4% (559), respectively. Nearly 50% (2,592) of accidents involved traffic-associated collisions, with approximately 25% (1,395) of those incidents involving a car, pick-up truck, or van. Helmet use was associated with fewer ventilator days (0.43 ± 3.16 vs. 0.67 ± 3.93, p = 0.0148) and higher GCS scores (13.98 ± 3.28 vs. 13.75 ± 3.52, p = 0.0141) compared to the non-helmeted group. However, ISS and total hospital days showed no statistically significant differences between the two groups.

Conclusion

This study demonstrates that helmet use among e-bike riders is associated with improved clinical outcomes, specifically higher GCS scores and fewer ventilator days. These findings reinforce the well-recognized benefits of helmet use across all age groups. Despite this, helmet compliance among e-bike riders remains low nationwide. Future research should examine long-term trends in e-bike incidents, hospital outcomes, and the impact of additional protective measures and regulatory policies. Continued investigation in this field is needed to identify injury patterns and guide public health initiatives aimed at enhancing rider safety.

## Introduction

In recent years, the United States has seen immense surges in the popularity of electric pedal-assisted bicycles (e-bikes). These bikes assume the standard appearance of conventional bicycles but are engineered with a motor that provides targeted power assistance to users while they are pedaling. This assistance greatly eases the physical efforts required while riding, thus opening the door to longer distances, difficult terrain, and ease while encountering large hills [1,2]. Access to these bikes has enabled many individuals to adopt more active and healthy lifestyles [3-6]. 

E-bikes serve several purposes such as commuting and recreation and are distributed into three federally recognized classes with variability between power, use of a throttle, and top speed. These classes provide clear standards for nationwide manufacturers [7]. As e-bike use continues to rise, so do concerns about rider safety, leading to evolving regulations and public health initiatives.

Given the increase in e-bike use nationwide, concern has been sparked regarding the safety associated with this method of travel. As this is a new emerging mode of transportation, statewide law and local government policies regarding their use tend to vary widely [8]. For example, California state law was recently updated to require all persons under the age of 17 on any class of e-bike to wear a helmet. This varies from Colorado state law, which requires helmets to be worn by all individuals under the age of 21 riding a class 3 e-bicycle but does not mention the requirement of helmets for other classes [9,10]. Additionally, public health initiatives aimed at reducing e-bike incidents adopt a variety of approaches, with some suggesting mandatory helmet use for all ages, and others even proposing licensing requirements for e-bike use, something that has been adopted in countries such as Germany and Japan. In some trail networks, local policies have been implemented to create offroad trail-specific restrictions for class 2 and class 3 e-bikes. 

Because high speeds up to 28 mph are encountered during e-bike travel, riders have a significant reduction in reaction time, thus exposing them to potentially greater incident likelihood [11]. Furthermore, riders in traffic heavy areas may be more susceptible to incidents from car crashes due to retrograde cycling, violating traffic lights, or lack of awareness of laws and traffic safety regulations [12-14].

E-bike injuries continually increase, with just 751 documented e-bike injuries in 2017 to 23,493 in 2022 [5]. While there is substantial evidence indicating the clear health benefits of bicycle use outside, there are concerns about health and safety that should be acknowledged. Given the improvement in helmet safety standards, we aim to determine the effectiveness of helmet use in reducing injury severity and improvement inhospital outcomes for those participating in these hobbies [15,16]. 

## Materials and methods

We conducted a retrospective analysis of patients treated at trauma centers across the United States from 2018 to 2023. Data were obtained from the National Trauma Data Standard (NTDS) as a de-identified dataset. Patients were eligible for inclusion if they sustained a traumatic injury within 14 days prior to hospital presentation [17]. Eligible cases were identified using the International Classification of Diseases, 10th Revision (ICD-10-CM) codes.

The NTDS dataset was screened for cases involving electric pedal-assisted bicycle incidents using 77 specific ICD-10 codes (Appendix). Due to limited case identification before 2022 NTDS, only data from 2022 and 2023 were included in the final analysis. After applying exclusion criteria, a total of 5,474 patients were analyzed. For each patient, demographic variables (age and sex), injury characteristics (Injury Severity Score [ISS], Glasgow Coma Scale [GCS]), and clinical outcomes (helmet use, hospital length of stay, use of ventilator and ventilator days) were extracted.  Data entries with missing or incomplete values for the variable being analyzed were excluded from the analysis.

Statistical analysis was conducted using Python 3.12.6 to assess demographic distribution (age and gender) as well as differences in clinical outcomes between helmeted and non-helmeted patients. Levene’s test was used to evaluate variance equality for helmet use comparisons across ventilator days, inpatient days, ISS, and GCS. Depending on variance assumptions, either Welch’s t-test (ventilator days, and GCS) or Student’s t-test (inpatient days, ISS) was performed, with an alpha value of 0.05 for all comparisons.

## Results

**Figure 1:**
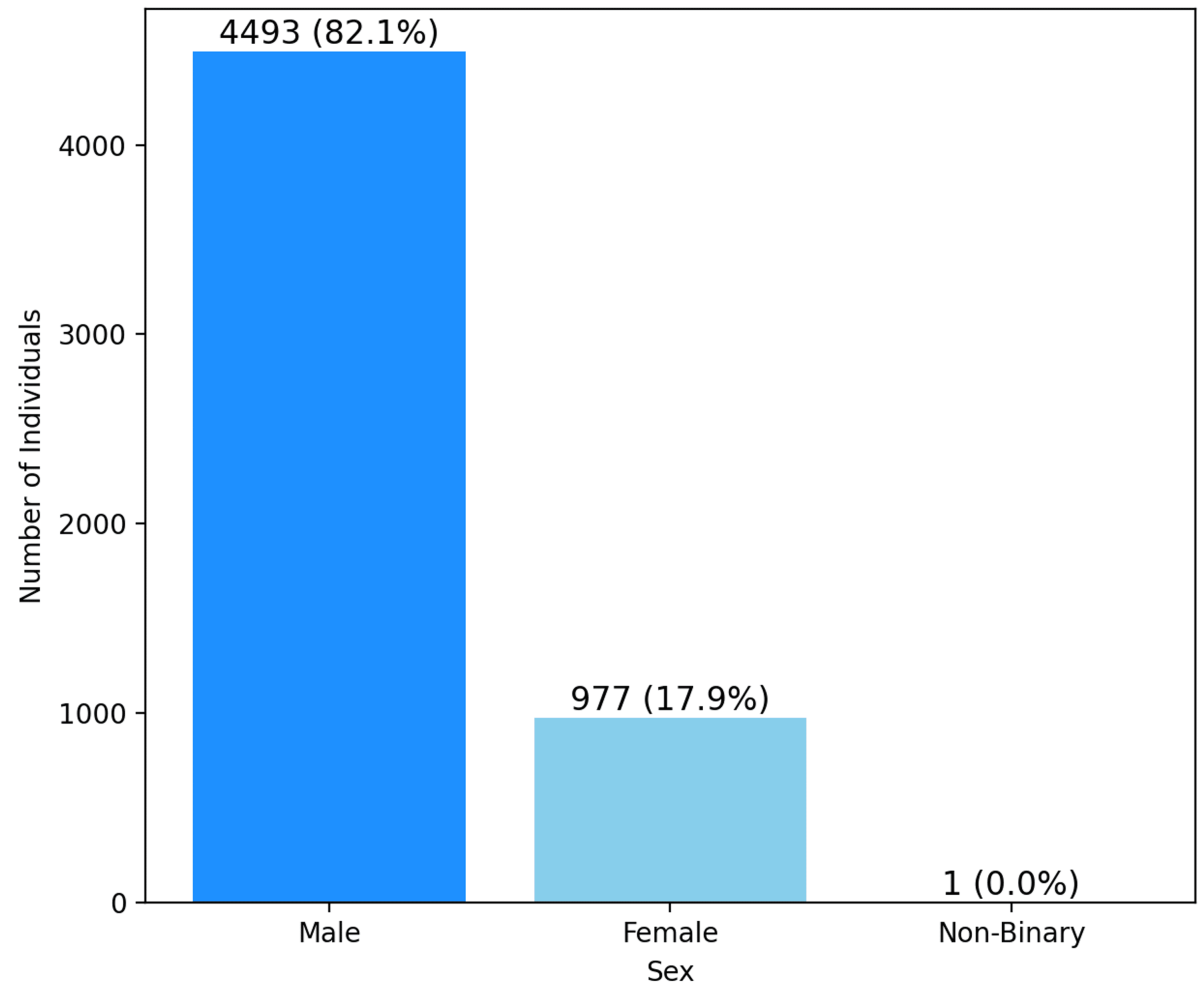
Visual representation of the total amount of e-bike crashes by sex

**Figure 2:**
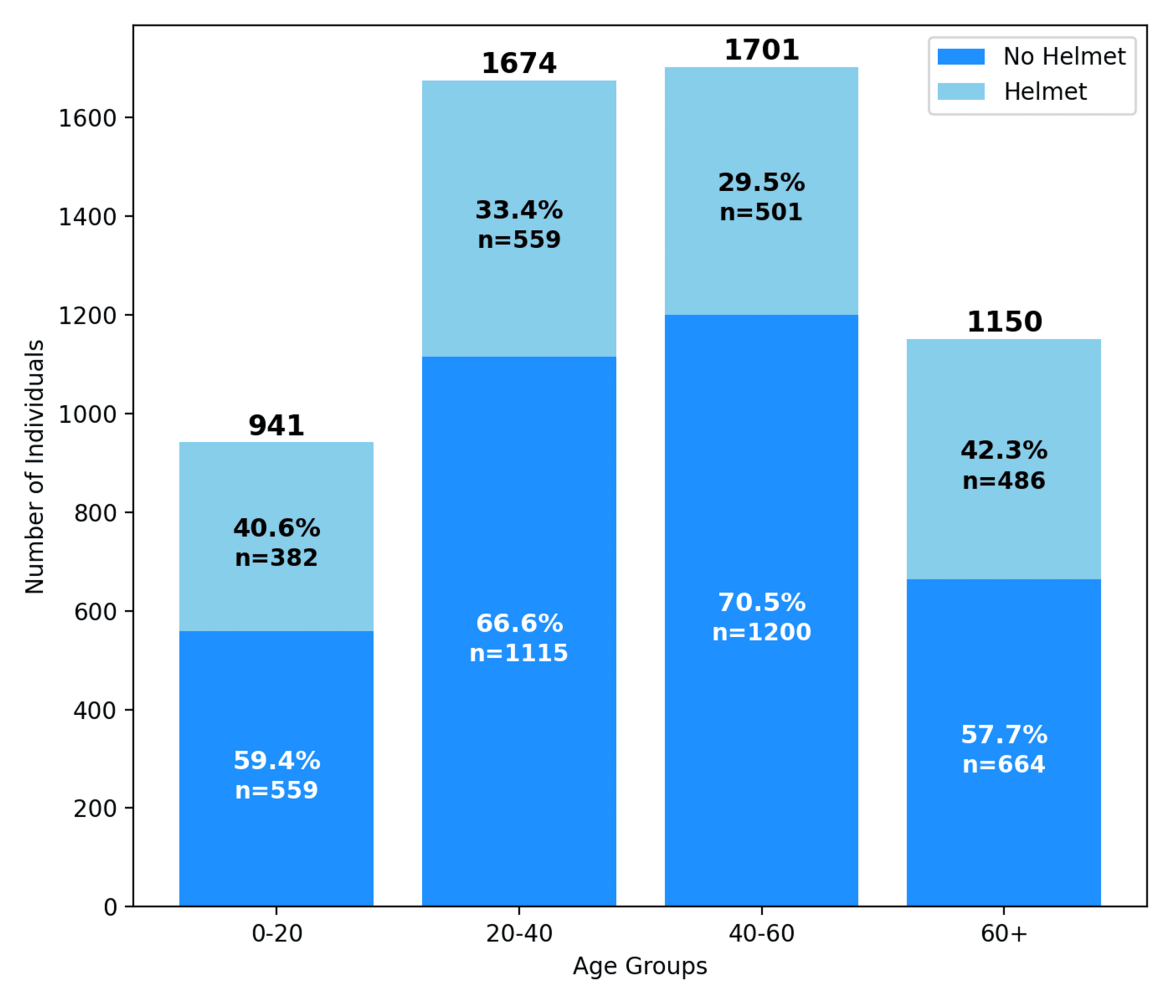
Schematic representation of the age groups involved in e-bike crashes and the percentage of those groups using helmets.

A total of 5,474 patients were included in this study; however, three were excluded from the mean age calculation due to missing data. The mean age of the remaining patients was 41.1 ± 19.2 years. The majority of patients (82%) were male (Figure 1). The distribution of e-bike accidents by age group showed that 941 (17.2%) individuals were between the ages of 0 and 20, 1,674 (30.6%) individuals were between 21 and 40, 1,701 (31.1%) individuals were between 41 and 60, and 1,150 (21.0%) individuals were 60 years or older (Figure 2). Eight individuals were excluded due to missing age data. The proportion of helmeted versus non-helmeted individuals was 382 (40.6%) to 559 (59.4%) in the 0- to 20-year-olds, 559 (33.4%) to 1,115 (66.6%) in 20- to 40-year-olds, 501 (29.5%) to 1,200 (70.5%) in 40-to 60-year-olds, and 486 (42.3%) to 664 (57.7%) in individuals over 60 (Figure 2).

**Figure 3:**
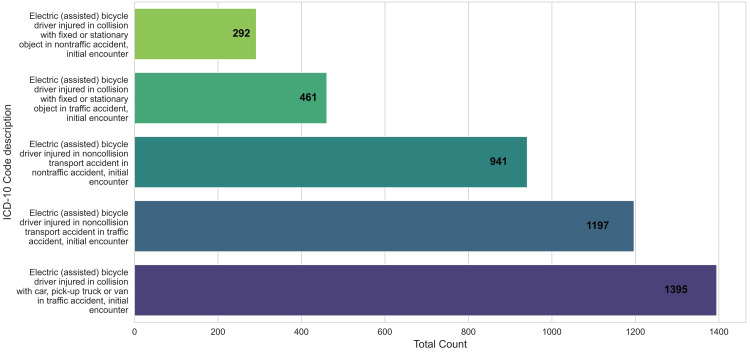
Visual display of the top five ICD-10 codes, including their descriptions and the number of reported cases from 2022 to 2023.

Additionally, the distribution of e-bike-related accidents demonstrated that almost 50% (2,592) of accidents involved a collision with a car, pick-up truck, or van in traffic accidents or non-collision transport accident in traffic (Figure 3).

Helmet use was analyzed in relation to ventilator days, inpatient length of stay, ISS, and GCS scores (Table 1). The mean number of ventilator days was lower in the helmeted group (0.43 ± 3.16) compared to the non-helmeted group (0.67 ± 3.93). Levene’s test indicated unequal variances (p = 0.0222), and Welch’s t-test showed a statistically significant difference (t = -2.4383, p = 0.0148), with point-biserial correlation analysis revealing a weakly negative correlation (r = -0.0309, p = 0.0222).

**Table 1:** Data obtained from statistical analysis between helmet and non-helmet uses across several NTDS criteria. GCS, Glasgow Coma Scale; ISS, Injury Severity Score *Statistically significant

Metric	Ventilator Days	Inpatient Days	ISS	GCS
Helmet group (mean ± SD)	0.43 ± 3.16	4.49 ± 6.73	10.01 ± 7.98	13.98 ± 3.28
No helmet group (mean ± SD)	0.67 ± 3.93	4.82 ± 9.56	10.17 ± 8.04	13.75 ± 3.52
T-test (t-statistic, p-value)	-2.4383, 0.0148	-1.3212, 0.1865	-0.7243, 0.4689	2.4552, 0.0141
Statistical significance	Significant (p < 0.05)*	Not significant	Not significant	Significant (p < 0.05)*

For inpatient length of stay, the helmeted group had a mean hospital stay of 4.49 ± 6.73 days, while the non-helmeted group had a slightly longer stay of 4.82 ± 9.56 days. Levene’s test indicated equal variances (p = 0.3997), and Student’s t-test found no statistically significant difference between the two groups (t = -1.3212, p = 0.1865). Similarly, point-biserial correlation analysis showed a weak negative correlation (r = -0.0179, p = 0.1865).

Regarding injury severity, the mean ISS was 10.01 ± 7.98 in the helmeted group and 10.17 ± 8.04 in the non-helmeted group. Levene’s test indicated equal variances (p = 0.1872), and Student’s t-test showed no statistically significant difference (t = -0.7243, p = 0.4689). The point-biserial correlation analysis also indicated a very weak negative correlation (r = -0.0098, p = 0.4689).

In contrast, helmet use was associated with higher GCS scores. The helmeted group had a mean GCS of 13.98 ± 3.28, while the non-helmeted group had a mean GCS of 13.75 ± 3.52. Levene’s test indicated unequal variances (p = 0.0163), and Welch’s t-test demonstrated a statistically significant difference (t = 2.4552, p = 0.0141). Point-biserial correlation analysis revealed a weak positive correlation (r = 0.0325, p = 0.0163), suggesting that helmeted riders had slightly better neurological outcomes.

## Discussion

The findings of this study highlight the impact of helmet use on injury severity among e-bike riders and emphasize the necessity of targeted safety interventions. The results indicated a significant correlation between helmet use and reduction in severe injuries, reinforcing existing literature that underscores the protective benefits associated with helmets in cycling-related accidents [18-20]. Given the increasing popularity of e-bikes, these findings are highly relevant to public health and the development and implementation of traffic regulation regarding this mode of transportation. 

One key observation from this study is the higher incidence of severe injuries among non-helmeted riders compared to those wearing helmets, as shown through overall GCS and ISS values. In parallel with previous research indicating positive effects of helmet use in traditional bike crashes, our findings suggest that helmets provide substantial protection against traumatic brain injuries and skull fractures for those using e-bikes [9-12,21]. One study suggested that the odds of sustaining a head injury while not wearing a helmet on an e-bike were 1.9 times higher than those wearing a helmet [21]. Given the increased speeds of e-bikes compared to traditional bicycles, it is possible that helmet use is even more crucial to reducing the injury severity of people involved in collisions.

Our analysis also revealed that over half of the e-bike-related accidents reported involved either collisions with cars, pick-up trucks, or vans in a traffic accident or a non-collision transport accident in traffic. These findings indicate potential risks associated with sharing the road with larger, faster vehicles, and the need to direct research into the root causes of these incidents. Some proposed causes may include limited visibility of e-bike riders, unsafe passing/failure to yield, or lack of dedicated bike lanes/road infrastructure for this growing mode of transportation [22]. Additionally, non-collision transport accidents in traffic accounted for 22% (1,167) of accidents, which may be attributed to loss of control due to road conditions, sudden braking, or swerving to avoid obstacles or mechanical failures/rider inexperience.

A literature review analyzing the relationship between e-bike incidents and behaviors in China and Europe suggested that most e-bike-related incidents are associated with risk riding behaviors, including overspeeding, running red lights, distracted riding, and going against traffic flow [23]. While countries such as Germany and the Netherlands have also conducted studies to identify the causes of e-bike-related accidents, research in the United States remains limited. Most research surrounding the topic of e-bikes are retrospective cross-sectional studies as opposed to naturalistic studies that observe rider behavior in real-world conditions [24-28]. Given the rapid rise in e-bike use, there is a pressing need for a more comprehensive, behavior-focused analysis to better understand the risks and subsequent safety interventions that can be implemented.

Another important aspect of this study is the demographic distribution of e-bike accidents and helmet use based on age and gender. Our analysis reveals clear distinctions in incident frequency between age groups. Although older adults represent a smaller proportion of total e-bike incidents, they may be more likely to experience greater injury severity (as reflected by higher ISS scores) and longer hospital stays. This may be attributed to age-related physiological factors such as decreased balance, reduced bone density, and slower healing capacity [29-31]. These factors could contribute to the higher average ISS and inpatient day values obtained in this study. With this in mind, future research may benefit from exploring age-related differences in recovery time and injury outcomes following e-bike-related trauma. Moreover, our results suggest that a disproportionate number of individuals involved in e-bike accidents were male, raising the question: Are men more likely to be involved in e-bike crashes, or is this simply a reflection of market demographics, where men make up a larger share of e-bike consumers?

Several studies have analyzed e-bike purchasing trends, with many of these suggesting that a greater proportion of e-bike owners are male [1,32,33]. Current research does not clearly define whether the higher incidence of male e-bike accidents is due solely to their greater market presence or if other factors such as risk-taking behaviors play a larger role. A variety of research supports the notion that males are more likely to engage in risk-taking behavior, which may explain their higher incidence of e-bike-related injuries [1,34]. Additionally, studies examining risk-taking tendencies among e-bike riders suggest that behavioral differences significantly influence crash risk [23]. Given this, it is possible that male riders are overrepresented in accident data not merely because they outnumber female riders but because they engage in riskier riding behaviors, particularly in recreational settings [35]. Future research should explore these factors in greater depth to determine whether this in fact is a cause of this disproportion and evaluate possible interventions if this is the case, such as risk-awareness training that could mitigate these disparities.

Regarding helmet use, our data indicate that younger riders (ages 0-20) and older individuals (60+) were more likely to wear helmets, yet across all age groups, non-helmet use remained more prevalent than helmet compliance. Understanding these patterns can help shape targeted education campaigns and policy interventions. For instance, in the age range of 0-20 years, there was a 59% (559) to 41% (382) split between non-helmet and helmet users. Given this, young or inexperienced riders may benefit from structured safety training programs that emphasize the importance of helmet compliance in hopes of fostering long-term safety habits [36].

Although many states have mandatory helmet laws, there remains significant opposition to wearing them [37]. This raises the question of whether alternative methods could improve compliance and, if so, how they can be effectively implemented to increase helmet use. Examining countries with high helmet compliance despite the absence of mandatory legislation may provide valuable insight. Reported interventions include targeted safety campaigns that emphasize personal responsibility and safety, as well as strong community engagement efforts, particularly those encouraging helmet use among younger cyclists [38]. These approaches may offer a model for improving helmet compliance in the United States without relying solely on legislation.

While this study provides valuable insights, certain limitations should be acknowledged. The reliance on self-reported data introduces potential biases, and the sample size may not be representative of all e-bike users. The NTDS data only factors into account the number of individuals who presented to the trauma bay and does not include all individuals who presented to the ED or those who received minimal injuries not necessitating ED evaluation. Furthermore, the type of injury sustained by individuals was not examined in this study. This could represent an additional avenue for research to determine how injury mechanism contributes to patient outcomes. Additionally, while e-bikes have been in use since the 1990s, they did not gain popularity until 2020 and did not see their incorporation into ICD codes until the 2023 fiscal year update. Additionally, the use of specific ICD-10 codes may not accurately represent the extent or specific cause of an accident as some ICD-10 codes may be more preferred than others when documenting these cases [39-41]. Lastly, it should also be noted that a variety of helmet styles are present in the current market. These include standard open-face road biking helmets, open-face mountain biking helmets, and full-face helmets. The NTDS data set does not provide a distinction between subtypes of helmets used in this patient population. As such, future research should be directed toward the distinctions between helmet classifications and their respective injury reduction patterns as there are large differences between injury outcomes [42]. We look forward to future iterations of ICD coding that provide distinction between helmet classes along with extensive analysis of e-bike injury patterns.

## Conclusions

Our analysis of the use of helmets with respect to e-bike incidents has generally supported the general literature findings. NTDS data from the years in question suggested an overall increased GCS score and fewer ventilator days for patients involved in e-bike incidents and wearing helmets. While acknowledging gaps in some data, these findings highlight the increasing prevalence of e-bike-related accidents and bring forth the need for further interdisciplinary research combining public health and transportation planning to develop comprehensive safety strategies for the growing e-bike population.

## Appendices

**Table 2:** ICD codes included in analysis [34]

Primary Code	Description
V20.01XA	Electric bike rider injured in collision with pedestrian or animal, initial encounter
V20.11XA	Electric bike rider injured in collision with pedal cycle, initial encounter
V20.21XA	Electric bike rider injured in collision with two- or three-wheeled motor vehicle, initial encounter
V20.31XA	Electric bike rider injured in collision with car, pick-up truck, or van, initial encounter
V20.41XA	Electric bike rider injured in collision with heavy transport vehicle or bus, initial encounter
V20.51XA	Electric bike rider injured in collision with railway train or railway vehicle, initial encounter
V20.91XA	Electric bike rider injured in unspecified traffic accident, initial encounter
V21.01XA	Electric (assisted) bicycle driver injured in collision with pedal cycle in nontraffic accident, initial encounter
V21.11XA	Electric (assisted) bicycle passenger injured in collision with pedal cycle in nontraffic accident, initial encounter
V21.21XA	Unspecified electric (assisted) bicycle rider injured in collision with pedal cycle in nontraffic accident
V21.31XA	Person boarding or alighting an electric (assisted) bicycle injured in collision with pedal cycle, initial encounter√ä
V21.41XA	Electric (assisted) bicycle driver injured in collision with pedal cycle in traffic accident, initial encounter
V21.51XA	Electric (assisted) bicycle passenger injured in collision with pedal cycle in traffic accident, initial encounter
V21.91XA	√äUnspecified electric (assisted) bicycle rider injured in collision with pedal cycle in traffic accident, initial encounter
V22.01XA	Electric (assisted) bicycle driver injured in collision with two- or three-wheeled motor vehicle in nontraffic accident, initial encounter
V22.11XA	Electric (assisted) bicycle passenger injured in collision with two- or three-wheeled motor vehicle in nontraffic accident, initial encounter
V22.21XA	Unspecified electric (assisted) bicycle rider injured in collision with two- or three-wheeled motor vehicle in nontraffic accident, initial encounter
V22.31XA	Person boarding or alighting an electric (assisted) bicycle injured in collision with two- or three-wheeled motor vehicle, initial encounter
V22.41XA	Electric (assisted) bicycle driver injured in collision with two- or three-wheeled motor vehicle in traffic accident, initial encounter
V22.51XA	Electric (assisted) bicycle passenger injured in collision with two- or three-wheeled motor vehicle in traffic accident, initial encounter
V22.91XA	Unspecified electric (assisted) bicycle rider injured in collision with two- or three-wheeled motor vehicle in traffic accident, initial encounter
V23.01XA	Electric (assisted) bicycle driver injured in collision with car, pick-up truck, or van in nontraffic accident, initial encounter
V23.11XA	Electric (assisted) bicycle passenger injured in collision with car, pick-up truck, or van in nontraffic accident, initial encounter
V23.21XA	Unspecified electric (assisted) bicycle rider injured in collision with car, pick-up truck, or van in nontraffic accident, initial encounter
V23.31XA	Person boarding or alighting an electric (assisted) bicycle injured in collision with car, pick-up truck, or van, initial encounter
V23.41XA	Electric (assisted) bicycle driver injured in collision with car, pick-up truck, or van in traffic accident, initial encounter
V23.51XA	Electric (assisted) bicycle passenger injured in collision with car, pick-up truck, or van in traffic accident, initial encounter
V23.91XA	Unspecified electric (assisted) bicycle rider injured in collision with car, pick-up truck, or van in traffic accident, initial encounter
V24.01XA	Electric (assisted) bicycle driver injured in collision with heavy transport vehicle or bus in nontraffic accident, initial encounter
V24.11XA	Electric (assisted) bicycle passenger injured in collision with heavy transport vehicle or bus in nontraffic accident, initial encounter
V24.21XA	Unspecified electric (assisted) bicycle rider injured in collision with heavy transport vehicle or bus in nontraffic accident, initial encounter
V24.31XA	Person boarding or alighting an electric (assisted) bicycle injured in collision with heavy transport vehicle or bus, initial encounter
V24.41XA	Electric (assisted) bicycle driver injured in collision with heavy transport vehicle or bus in traffic accident, initial encounter
V24.51XA	Electric (assisted) bicycle passenger injured in collision with heavy transport vehicle or bus in traffic accident, initial encounter
V24.91XA	Unspecified electric (assisted) bicycle rider injured in collision with heavy transport vehicle or bus in traffic accident, initial encounter
V25.01XA	Electric (assisted) bicycle driver injured in collision with railway train or railway vehicle in nontraffic accident, initial encounter
V25.11XA	Electric (assisted) bicycle passenger injured in collision with railway train or railway vehicle in nontraffic accident, initial encounter
V25.21XA	Unspecified electric (assisted) bicycle rider injured in collision with railway train or railway vehicle in nontraffic accident, initial encounter
V25.31XA	Person boarding or alighting an electric (assisted) bicycle injured in collision with railway train or railway vehicle, initial encounter
V25.51XA	Electric (assisted) bicycle passenger injured in collision with railway train or railway vehicle in traffic accident, initial encounter
V25.91XA	Unspecified electric (assisted) bicycle rider injured in collision with railway train or railway vehicle in traffic accident, initial encounter
V26.01XA	Electric (assisted) bicycle driver injured in collision with other nonmotor vehicle in nontraffic accident, initial encounter
V26.11XA	Electric (assisted) bicycle passenger injured in collision with other nonmotor vehicle in nontraffic accident, initial encounter
V26.21XA	Unspecified electric (assisted) bicycle rider injured in collision with other nonmotor vehicle in nontraffic accident, initial encounter
V26.31XA	Person boarding or alighting an electric (assisted) bicycle injured in collision with other nonmotor vehicle, initial encounter
V26.41XA	Electric (assisted) bicycle driver injured in collision with other nonmotor vehicle in traffic accident, initial encounter
V26.51XA	Electric (assisted) bicycle passenger injured in collision with other nonmotor vehicle in traffic accident, initial encounter
V26.91XA	Unspecified electric (assisted) bicycle rider injured in collision with other nonmotor vehicle in traffic accident, initial encounter
V27.01XA	Electric (assisted) bicycle driver injured in collision with fixed or stationary object in nontraffic accident, initial encounter
V27.11XA	Electric (assisted) bicycle passenger injured in collision with fixed or stationary object in nontraffic accident, initial encounter
V27.21XA	Unspecified electric (assisted) bicycle rider injured in collision with fixed or stationary object in nontraffic accident, initial encounter
V27.31XA	Person boarding or alighting an electric (assisted) bicycle injured in collision with fixed or stationary object, initial encounter
V27.41XA	Electric (assisted) bicycle driver injured in collision with fixed or stationary object in traffic accident, initial encounter
V27.51XA	Electric (assisted) bicycle passenger injured in collision with fixed or stationary object in traffic accident, initial encounter
V28.01XA	Electric (assisted) bicycle driver injured in noncollision transport accident in nontraffic accident, initial encounter
V28.11XA	Electric (assisted) bicycle passenger injured in noncollision transport accident in nontraffic accident, initial encounter
V28.21XA	Unspecified electric (assisted) bicycle rider injured in noncollision transport accident in nontraffic accident, initial encounter
V28.31XA	Person boarding or alighting an electric (assisted) bicycle injured in noncollision transport accident, initial encounter
V28.41XA	Electric (assisted) bicycle driver injured in noncollision transport accident in traffic accident, initial encounter
V28.51XA	Electric (assisted) bicycle passenger injured in noncollision transport accident in traffic accident, initial encounter
V28.91XA	Unspecified electric (assisted) bicycle rider injured in noncollision transport accident in traffic accident, initial encounter
V29.001A	Electric (assisted) bicycle driver injured in collision with unspecified motor vehicles in nontraffic accident, initial encounter
V29.091A	Electric (assisted) bicycle driver injured in collision with other motor vehicles in nontraffic accident, initial encounter
V29.101A	Electric (assisted) bicycle passenger injured in collision with unspecified motor vehicles in nontraffic accident, initial encounter
V29.191A	Electric (assisted) bicycle passenger injured in collision with other motor vehicles in nontraffic accident, initial encounter
V29.201A	Unspecified electric (assisted) bicycle rider injured in collision with unspecified motor vehicles in nontraffic accident, initial encounter
V29.291A	Unspecified electric (assisted) bicycle rider injured in collision with other motor vehicles in nontraffic accident, initial encounter
V29.31XA	Electric (assisted) bicycle (driver) (passenger) injured in unspecified nontraffic accident, initial encounter
V29.401A	Electric (assisted) bicycle driver injured in collision with unspecified motor vehicles in traffic accident, initial encounter
V29.491A	Electric (assisted) bicycle driver injured in collision with other motor vehicles in traffic accident, initial encounter
V29.501A	Electric (assisted) bicycle passenger injured in collision with unspecified motor vehicles in traffic accident, initial encounter
V29.591A	Electric (assisted) bicycle passenger injured in collision with other motor vehicles in traffic accident, initial encounter
V29.601A	Unspecified electric (assisted) bicycle rider injured in collision with unspecified motor vehicles in traffic accident, initial encounter
V2.9691A	Unspecified electric (assisted) bicycle rider injured in collision with other motor vehicles in traffic accident, initial encounter
V29.811A	Electric (assisted) bicycle rider (driver) (passenger) injured in transport accident with military vehicle, initial encounter
V29.881A	Electric (assisted) bicycle rider (driver) (passenger) injured in other specified transport accidents, initial encounter
V29.91XA	Electric (assisted) bicycle rider (driver) (passenger) injured in unspecified traffic accident, initial encounter
